# Sensitivity and Specificity of Medial Temporal Lobe Visual Ratings and Multivariate Regional MRI Classification in Alzheimer's Disease

**DOI:** 10.1371/journal.pone.0022506

**Published:** 2011-07-21

**Authors:** Eric Westman, Lena Cavallin, J-Sebastian Muehlboeck, Yi Zhang, Patrizia Mecocci, Bruno Vellas, Magda Tsolaki, Iwona Kłoszewska, Hilkka Soininen, Christian Spenger, Simon Lovestone, Andrew Simmons, Lars-Olof Wahlund, for the AddNeuroMed consortium

**Affiliations:** 1 Department of Neurobiology, Care Sciences and Society, Karolinska Institutet, Stockholm, Sweden; 2 Department of Clinical Science, Intervention and Technology, Karolinska Institutet, Stockholm, Sweden; 3 Department of Radiology, Karolinska University Hospital, Stockholm, Sweden; 4 Institute of Gerontology and Geriatrics, University of Perugia, Perugia, Italy; 5 INSERM U 558, University of Toulouse, Toulouse, France; 6 Aristotle University of Thessaloniki, Thessaloniki, Greece; 7 Medical University of Lodz, Lodz, Poland; 8 University of Eastern Finland and University Hospital of Kuopio, Kuopio, Finland; 9 King's College London, Institute of Psychiatry, London, United Kingdom; 10 NIHR Biomedical Research Centre for Mental Health, London, United Kingdom; Federal University of Rio de Janeiro, Brazil

## Abstract

**Background:**

Visual assessment rating scales for medial temporal lobe (MTL) atrophy have been used by neuroradiologists in clinical practice to aid the diagnosis of Alzheimer's disease (AD). Recently multivariate classification methods for magnetic resonance imaging (MRI) data have been suggested as alternative tools. If computerized methods are to be implemented in clinical practice they need to be as good as, or better than experienced neuroradiologists and carefully validated. The aims of this study were: (1) To compare the ability of MTL atrophy visual assessment rating scales, a multivariate MRI classification method and manually measured hippocampal volumes to distinguish between subjects with AD and healthy elderly controls (CTL). (2) To assess how well the three techniques perform when predicting future conversion from mild cognitive impairment (MCI) to AD.

**Methods:**

High resolution sagittal 3D T1w MP-RAGE datasets were acquired from 75 AD patients, 101 subjects with MCI and 81 CTL from the multi-centre AddNeuroMed study. An automated analysis method was used to generate regional volume and regional cortical thickness measures, providing 57 variables for multivariate analysis (orthogonal partial least squares to latent structures using seven-fold cross-validation). Manual hippocampal measurements were also determined for each subject. Visual rating assessment of MTL atrophy was performed by an experienced neuroradiologist according to the approach of Scheltens et al.

**Results:**

We found prediction accuracies for distinguishing between AD and CTL of 83% for multivariate classification, 81% for the visual rating assessments and 89% for manual measurements of total hippocampal volume. The three different techniques showed similar accuracy in predicting conversion from MCI to AD at one year follow-up.

**Conclusion:**

Visual rating assessment of the MTL gave similar prediction accuracy to multivariate classification and manual hippocampal volumes. This suggests a potential future role for computerized methods as a complement to clinical assessment of AD.

## Introduction

Dementia is the third most common cause of death in society today, exceeded only by cancer and cardiovascular disorders and Alzheimer's disease (AD) is the most common form of dementia. Biomarkers of AD based on non-invasive *in vivo* methods are highly desirable for diagnosis, monitoring disease progression and evaluating disease-modifying treatment strategies. An ideal biomarker would detect a fundamental feature of AD neuropathology, be diagnostically sensitive and specific, and produce accurate and reproducible results [1]. Magnetic Resonance Imaging (MRI), Positron Emission Tomography (PET), and cerebrospinal fluid (CSF) measures all allow different aspects of AD pathology to be studied. The new suggested research criterion for AD is centered on a clinical core of early and significant episodic memory impairment and at least one abnormal biomarker from MRI, PET and CSF [2] and the new suggested diagnostic criterion also utilize biomarkers [3].

Several groups including our own have proposed the use of multivariate techniques for analyzing multiple regional measures from MRI to aid diagnosis of AD and to predict future conversion from the prodromal stages of the disease often referred to as mild cognitive impairment (MCI) to AD. Previous studies have shown that computerized methods give high prediction accuracies when distinguishing between patient groups [4], [5], [6], [7]. If computerized methods based on MRI are to be useful in clinical practice then they will need to be as good as or better than experienced neuroradiologists. Klöppel et al. previously compared the diagnostic accuracy of a computerized method (support vector machines (SVM)) with radiological expertise, concluding that SVM gives comparable results to a well-trained neuroradiologist [8]. The study had the strength of using neuropathologically confirmed images, however they did not use a validated and widely used clinical rating scale and they studied relatively small cohorts.

We recently used the multivariate method orthogonal partial least squares to latent structures (OPLS) with multiple regional volumes and cortical thickness measures as input to investigate patterns of atrophy and prediction accuracy in two large multicenter cohorts (AddNeuroMed and the Alzheimer's Disease Neuroimaging Initiative (ADNI)). The study included over a thousand patients and we found prediction accuracies between 83–87% when discriminating between AD and controls [9].

The aim of the current study is to compare the performance of the OPLS multivariate technique with that of an experienced neuroradiologist using data from the AddNeuroMed cohort (a part of InnoMed, (Innovative Medicines in Europe), an Integrated Project funded by the European Union Sixth Framework programme) [10], [11]. The method used for visual rating assessment is the well established Scheltens method which uses a five point scale to grade atrophy in the medial temporal lobe [12]. To our knowledge this is the first comparison of the Scheltens visual rating scale for assessment of AD with a computerized method. Additionally our study uses a substantially larger cohort than the earlier study by Klöppel et al, and we also include subjects with mild cognitive impairment. For further evaluation we also aimed to compare the visual rating assessment with manual hippocampal measures. Manual measures have been used for AD diagnosis in research for many years and this region is one part of the visual rating assessment protocol described by Scheltens et al. However, manual hippocampal measurements are time consuming and not feasible in clinical practice. The different approaches were compared in two steps, firstly by distinguishing between AD and controls, and secondly by assessing how well the approaches predicted conversion of MCI subjects at baseline to AD at one year follow-up.

## Materials and Methods

### Ethics Statement

Written consent was obtained where the research participant had capacity, and in those cases where dementia compromised capacity then assent from the patient and written consent from a relative, according to local law and process, was obtained. This study was approved by ethical review boards in each participating country (local ethical review board at University of Perugia, University of Toulouse, Aristotle University of Thessaloniki, Medical University of Lodz, University of Eastern Finland and University Hospital of Kuopio and King's College London).

### Study data and inclusion and diagnostic criteria

All patients originated from the AddNeuroMed project, part of InnoMed (Innovative Medicines in Europe), a European Union program designed to make drug discovery more efficient. The project is designed to develop and validate novel surrogate markers in Alzheimer's disease (AD) and includes a human neuroimaging strand [13], [14] which combines MRI data with other biomarkers and clinical data. Data was collected from six different sites across Europe; University of Kuopio, Finland, University of Perugia, Italy, Aristotle University of Thessaloniki, Greece, King's College London, United Kingdom, University of Lodz, Poland and University of Toulouse, France. MRI images from a total of 252 subjects were included in this study; 75 AD patients, 101 MCI patients and 81 healthy controls. Demographics of the cohort are given in Table 1. All AD and MCI subjects were recruited from local memory clinics of the six participating sites while the control subjects were recruited from non-related members of the patient's families, caregiver's relatives or social centres for the elderly. The inclusion and exclusion criteria were as follows.

**Table 1:** Subject characteristics.

	AddNeuroMed		
	CTL	MCI	AD
**Number**	81	101	75
**Female/Male**	45/36	52/49	50/25
**Age**	73.6±6.3	74.0±5.8	74.2±6.0
**Education**	11.0±4.8	8.7±4.3	8.3±4.2
**MMSE**	29.0±1.2	27.2±1.6	21.3±4.6
**CDR**	0	0.5	1.1±0.5
**ADAS1**	3.4±1.5	5.3±1.2	6.6±1.5

Data are represented as mean ± standard deviation. AD = Alzheimer's disease, MCI = Mild Cognitive Impairment, CTL = healthy control, Education in years, MMSE = Mini Mental State Examination, ADAS1 = Word list non-learning (mean), CDR = Clinical Dementia Rating.

#### Alzheimer's disease

*Inclusion criteria*: 1) ADRDA/NINCDS and DSM- IV criteria for probable Alzheimer's disease. 2) Mini Mental State Examination score range between 12 and 28. 4) Age 65 years or above. *Exclusion criteria*: 1) Significant neurological or psychiatric illness other than Alzheimer's disease. This would exclude patients with vascular dementia or large infarcts. 2) Significant unstable systematic illness or organ failure. All AD subjects had a Clinical Dementia Rating (CDR) scale score of 0.5 or above.

#### Mild Cognitive Impairment and Controls

*Inclusion criteria*: 1) Mini Mental State Examination score range between 24 and 30. 2) Geriatric Depression Scale score less than or equal to 5. 3) Age 65 years or above. 4) Medication stable. 5) Good general health. *Exclusion criteria*: 1) Meet the DSM- IV criteria for Dementia. 2) Significant neurological or psychiatric illness other than Alzheimer's disease. 3) Significant unstable systematic illness or organ failure. The distinction between MCI and controls was based on two criteria: 1) subject scores 0 on Clinical Dementia Rating Scale = control. 2) Subject scores 0.5 on Clinical Dementia Rating scale = MCI. For the MCI subjects it was preferable that the subject and informant reported occurrence of memory problems.

CDR, Mini-Mental State, and CERAD Cognitive Battery were assessed for each subject. The CERAD Cognitive Battery was replaced with the Alzheimer's Disease Assessment Scale (ADAS–Cog) for the AD subjects. This cognitive test battery is specially designed for AD trials [15]. Both the ADAS-cog and the CERAD battery use the same 10-word recall task. The only difference is that the scoring is inverted. The mean number of words not recalled in the CERAD word list immediate recall task was calculated. The variable obtained was named ADAS1, corresponding to the first subtest of ADAS-Cog. This was performed to obtain comparable measures between groups.

### MRI

Data acquisition for the AddNeuroMed study was designed to be compatible with the Alzheimer Disease Neuroimaging Initiative (ADNI) [16]. The imaging protocol for both studies included a high resolution sagittal 3D T1-weighted MPRAGE volume (voxel size 1.1×1.1×1.2 mm^3^) and axial proton density/T2-weighted fast spin echo images. The MPRAGE volume was acquired using a custom pulse sequence specifically designed for the ADNI study to ensure compatibility across scanners [16]. Full brain and skull coverage was required for both of the latter datasets and detailed quality control carried out on all MR images from both studies according to the AddNeuroMed quality control procedure [13], [14].

### Regional volume segmentation and cortical thickness parcellation

**Figure 1:**
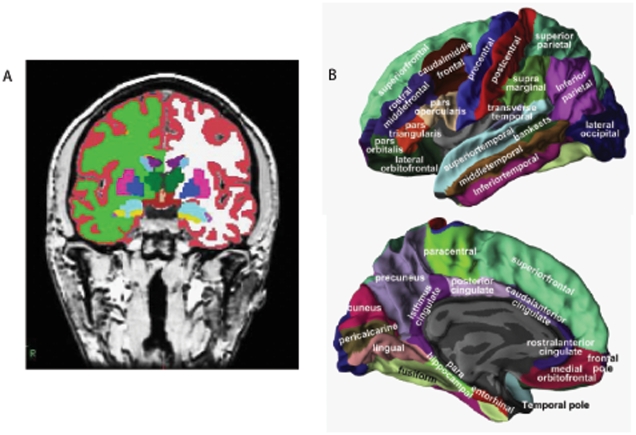
Representations of ROIs included as candidate input variables in the multivariate OPLS model. (A) Regional volumes. (B) Regional cortical thickness measures.

We utilized a pipeline, developed by Fischl and Dale which produces regional cortical thickness and volumetric measures (Figure 1). Cortical reconstruction and volumetric segmentation includes removal of non-brain tissue using a hybrid watershed/surface deformation procedure [17], automated Talairach transformation, segmentation of the subcortical white matter and deep grey matter volumetric structures (including hippocampus, amygdala, caudate, putamen, ventricles) [17], [18], [19] intensity normalization [20], tessellation of the grey matter white matter boundary, automated topology correction [21], [22], and surface deformation following intensity gradients to optimally place the grey/white and grey/cerebrospinal fluid borders at the location where the greatest shift in intensity defines the transition to the other tissue class [23], [24], [25]. Once the cortical models are complete, registration to a spherical atlas takes place which utilizes individual cortical folding patterns to match cortical geometry across subjects [26]. This is followed by parcellation of the cerebral cortex into units based on gyral and sulcal structure [27], [28]. Left and right sided volumes and thicknesses were averaged. The regional cortical thickness was measured from 34 areas and the regional volumes were measured from 23 areas. All volumetric measures from each subject were normalized by the subject's intracranial volume. This segmentation approach has been used for multivariate classification of Alzheimer's disease and healthy controls [7], neuropsychological-image analysis [29], [30], [31], imaging-genetic analysis [32], [33] and biomarker discovery [34].

### Visual rating assessment for medial temporal lobe atrophy

**Figure 2:**
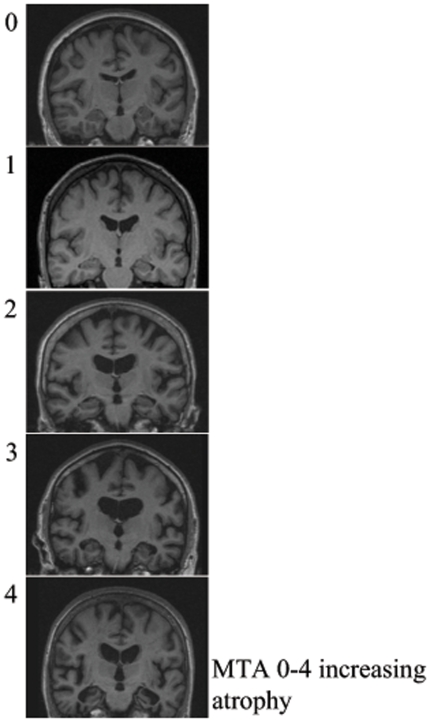
Visual assessment of the medial temporal lobe atrophy was performed on a single MR-slice posterior to the amygdala and the mamillary bodies. The was positioned so the hippocampus, the pons and the cerebral peduncles were all visible. The visual assessment included hippocampus proper, dentate gyrus, subiculum, parahippocampal gyrus, entorhinal cortex and surrounding CSF spaces such as temporal horn and choroid fissure. The right and left side were rated separately. Scores range from 0 (no atrophy) to 4 (end stage atrophy).

The 3D T1-weighted MPRAGE images were reoriented to an oblique coronal orientation perpendicular to the AC-PC (anterior commisure - posterior commisure) line suitable for both volumetric and visual assessment. Visual assessment of the medial temporal lobe atrophy was performed on a single MR-slice posterior to the amygdala and the mamillary bodies positioned such that the hippocampus, the pons and the cerebral peduncles are all covered by the slice. The rating scheme used here was first proposed by Scheltens et al. [12] and is based on a visual estimation of volume of the medial temporal lobe. The visual assessment includes hippocampus proper, dentate gyrus, subiculum, parahippocampal gyrus, entorhinal cortex and surrounding CSF spaces such as the temporal horns and choroid fissure. The right and left sides are rated separately. Scores range from 0 (no atrophy) to 4 (end stage atrophy) as detailed in Table 2 and Figure 2. For subjects <75 years, a MTA score of 2 or more is considered abnormal, while for subjects >75 years, a MTA score of 3 or more is considered abnormal (http://www.radiologyassistant.nl/en/43dbf6d16f98d). The rater (LC) was blinded to diagnosis, gender and age. Intra-rater reliability of the visual assessment of the medial temporal lobe atrophy was tested in 100 randomly selected subjects by repeated assessment with an interval of one week. Intra-rater reliability was 0.81 on right side and on left side 0.78. Weighted kappa was 0.93 on both sides.

**Table 2:** Visual rating of the medial temporal lobe.

Scale	Width of Choroid fissure	Width of temporal horn	Hippocampal thickness
**0**	N	N	N
**1**	↑	N	N
**2**	↑↑	↑	↓
**3**	↑↑↑	↑↑	↓↓
**4**	↑↑↑	↑↑↑	↓↓↓

Scheltens et al., 1992.

### Manual segmentation of hippocampus

Manual measurements of hippocampal volume were performed on a HERMES workstation (Nuclear Diagnostics, Stockholm, Sweden). Each measurement was performed with constant parameters by a neuroradiologist (YZ) who was blinded to clinical information. A ROI tool was used within the HERMES Multimodality software package, to manually delineate the hippocampal formation using previously defined anatomical landmarks [35]. Intra-rater reliability of the measurements was tested in 15 randomly selected subjects by repeated measurements with an interval of one month. The intra class correlation coefficients (ICC) of the measurements were >0.93. The total hippocampal volume from each subject was normalized by the subjects' intracranial volume.

### Multivariate data analysis

MRI measures were analyzed using orthogonal partial least squares to latent structures (OPLS) [6], [7], [36], [37], [38], [39], a supervised multivariate data analysis method included in the software package SIMCA (Umetrics AB, Umea, Sweden). A very similar method, partial least square to latent structures (PLS) has previously been used in several studies to analyze MR-data [40], [41], [42], [43], [44]. OPLS and PLS give the same predictive accuracy, but the advantage of OPLS is that the model created to compare groups is rotated, which means that the information related to class separation is found in the first component of the model, the predictive component. The other orthogonal components in the model, if any, relate to variation in the data not connected to class separation. Focusing the information related to class separation on the first component makes data interpretation easier [39].

Pre-processing was performed using mean centring and unit variance scaling. Mean centring improves the interpretability of the data, by subtracting the variable average from the data. By doing so the data set is repositioned around the origin. Large variance variables are more likely to be expressed in modeling than low variance variables. Consequently, unit variance scaling was selected to scale the data appropriately. This scaling method calculates the standard deviation of each variable. The inverse standard deviation is used as a scaling weight for each MR-measure.

The results from the OPLS analysis are visualized in a scatter plot by plotting the predictive component, which contains the information related to class separation. Components are vectors, which are linear combinations of partial vectors and are dominated by the input variables (x). The first and second components are by definition orthogonal to each other and span the projection plane of the points. Each point in the scatter plot represents one individual subject. The predictive component receives a Q^2^(Y) value that describes its statistical significance for separating groups. Q^2^(Y) values >0.05 are regarded as statistically significant [45], where
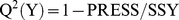
(1)where PRESS (predictive residual sum of squares) = Σ(y_actual_−y_predicted_)^2^ and SSY is the total variation of the Y matrix after scaling and mean centring [45]. Q^2^(Y) is the fraction of the total variation of the Ys (expected class values) that can be predicted by a component according to cross validation (CV). Cross validation is a statistical method for validating a predictive model which involves building a number of parallel models. These models differ from each other by leaving out a part of the data set each time. The data omitted is then predicted by the respective model. In this study we used seven fold cross-validation, which means that 1/7th of the data is omitted for each cross-validation round. Data is omitted once and only once. Variables were plotted according to their importance for the separation of groups. The plot shows the MRI measures and their corresponding jack-knifed confidence intervals. Jack-knifing is used to estimate the bias and standard error. Measures with confidence intervals that include zero have low reliability [39]. Covariance is plotted on the y-axis, where
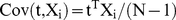
(2)where t is the transpose of the score vector t in the OPLS model, i is the centered variable in the data matrix X and N is the number of variables [39]. A measure with high covariance is more likely to have an impact on group separation than a variable with low covariance. MRI measures below zero in the scatter plot have lower values in controls compared to AD subjects, while MRI measures above zero are higher in controls compared to AD subjects in the model.

Altogether 57 variables were used for OPLS analysis. No feature selection was performed, meaning all measured variables were included in the analysis. A model containing age was also created to test if there were any significant differences between the diagnostic groups in relation to the variable. We investigated whether age would increase the predictive power of the models using it as an x-variable.

Sensitivity and specificity were calculated from the cross-validated prediction values of the OPLS models and for the visual assessment. Finally, the positive and negative likelihood ratios (LR+ = sensitivity/(100−specificity) and LR− = (100−sensitivity)/specificity) were calculated. A positive likelihood ratio between 5–10 or a negative likelihood ratio between 0.1–0.2 increases the diagnostic value in a moderate way, while a value above 10 or below 0.1 significantly increases the diagnostic value of the test [46].

Finally the AD vs. CTL models were used as training sets to investigate how well they could predict conversion from MCI to AD after one year follow-up and how they compare to the visual assessment. To easily compare the performance of the three methods we also calculated the sensitivity (MCI-c predicted as AD) at a fixed specificity (MCI-s predicted as CTL). We set the specificity for all three methods for this comparison to that of the visual assessment since this can not be changed and recalculated the sensitivity and specificity for the other two methods.

## Results

### Subject cohort

252 subjects were included in this study: 75 AD patients, 101 MCI patients and 81 control subjects. Using age as an x-variable in the OPLS models did not have any effect on the predictive power of the models separating the groups when all image variables were included. Therefore, age was excluded from further analysis. All MRI volumetric measures were normalised by dividing by each subject's intracranial volume. As expected, performance on the MMSE, CDR and ADAS1 was poorest among AD patients and best among controls (Table 1). The MCI group had scores between the AD and the control groups (Table 1).

### OPLS modelling and quality

Two models were created using (1) total hippocampal volume (2) automated regional volume and cortical thickness measures to compare AD vs. controls. The first model using the total manual hippocampal volume accounted for 100% of the variance of the original data (R^2^(X)) and its' cross validated predictability, Q^2^(Y) = 0.61. The second model using regional MRI measures resulted in one predictive component with R^2^(X) = 60% and cross validated predictability Q^2^(Y) = 0.45.

### Classification accuracy of the different techniques

**Figure 3:**
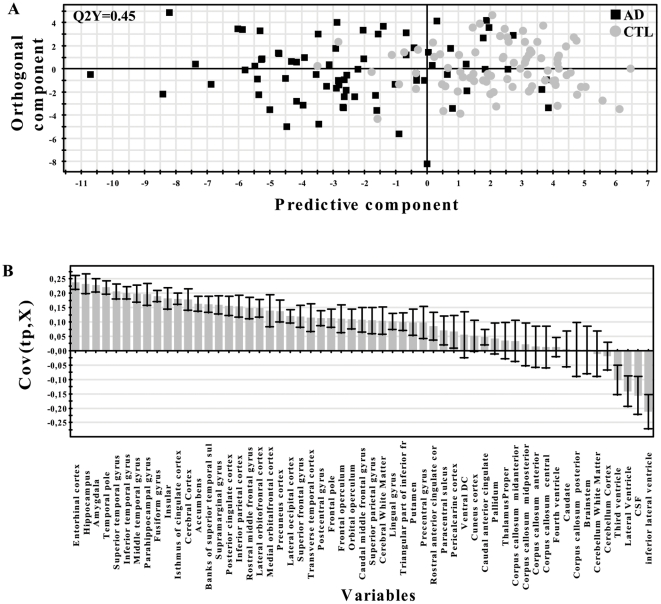
OPLS cross validated score plots and MRI measures of importance for the separation between AD and CTL. (A) The scatter plot visualises group separation and the predictability of the AD vs. CTL model. Each black square represents an AD subject and each gray circle a control subject. Control subjects to the left of zero and AD subjects to the right of zero are falsely predicted. Q^2^(Y)>0.05 (statistically significant model). (B) Measures above zero have a larger value in controls compared to AD and measures below zero have a lower value in controls compared to AD. A measure with a high covariance is more likely to have an impact on group separation than a measure with a low covariance. Measures with jack knifed confidence intervals that include zero have low reliability.

The separation between patients with AD and controls and the predictive power of the models Q^2^(Y) can be seen in Figure 3A using automated regional volume and cortical thickness measures as input. As can be observed there is a distinct separation between AD and controls. This model resulted in a prediction accuracy of 82.7% (accuracy, sensitivity, specificity, positive likelihood ratio and negative likelihood ratio given in Table 3). Figure 3B illustrates the variables of importance for the distinction between the two groups. The pattern of atrophy, including hippocampus, amygdala and entorhinal cortex among other temporal lobe regions, together with volume measures of CSF is as expected very similar to previous analyses of the AddNeuroMed cohort using regional MRI measures and an OPLS model [9]. Visual rating assessment using the Scheltens scale resulted in a prediction accuracy of 80.8%. Finally, total hippocampal volume yielded a prediction accuracy of 89.1%. The best predictive result was obtained from manual hippocampal measures closely followed by the automated image pipeline with OPLS and lastly the visual rating assessment.

**Table 3:** Sensitivity/specificity and likelihood ratios for the different methods.

	Sensitivity	Specificity	Accuracy	LR+	LR−
**Visual assessment**	78.7 (68.1–86.4)	82.7 (73.1–89.4)	80.8 (73.9–86.2)	4.6 (2.8–7.4)	0.26 (0.16–0.40)
**Fischl and Dale**	77.3(66.7–85.3)	87.7 (78.7–93.2)	82.7 (75.6–87.8)	6.3 (3.5–11.3)	0.26 (0.17–0.40)
**Manual outlining**	93.3 (85.3–97.1)	85.2(75.9–91.3)	89.1 (83.2–93.1)	6.3(3.7–10.7)	0.08 (0.03–0.18)

Confidence intervals within parentheses, LR+ = positive likelihood ratio and LR− = negative likelihood ratio.

### Predicting conversion from MCI at baseline to AD at one year follow-up

Finally, we wanted to investigate how the three different approaches would predict conversion from MCI at baseline to AD at one year clinical follow-up. All MCI subjects were classified as either AD or control like using OPLS models (AD vs. CTL). The visual assessment for the MCI subjects were performed in the same way, using the same cut offs as described previously, resulting in an assessment of abnormal or normal brain changes with respect to age. The results are shown in Table 4 demonstrating that 68% of the MCI converters (MCI-c) were classified as more AD-like and 68% of the MCI stable (MCI-s) classified as more control-like at baseline using visual rating assessment. Using automated regional MRI measures as input to the OPLS model, 74% of the MCI-c subjects who converted to AD at one year follow-up were predicted as more AD-like and 70% of the MCI-s were predicted as more control-like at baseline. For the total hippocampal volume, 79% of the MCI-c subjects who converted to AD at one year follow-up were predicted as more AD-like and 54% of the MCI-s were predicted as more control-like. When we set the specificity (MCI-s predicted as CTL) to a fixed value (the specificity of the visual assessment) to make the comparison of the methods easier, the results were slightly altered (Table 4). The best results were obtained from the OPLS model with automated regional MRI measures as input, with 79% of MCI-c subjects who converted to AD at one year follow-up predicted as more AD-like, compared to 68% for the manual hippocampal volumes and Scheltens visual assessment rating.

**Table 4:** MCI prediction.

Method	Number	AD-like	CTL-like
Visual assessment converters	19	68% (13)	32% (6)
Fischl and Dale converters	19	74% (14)	26% (5)
Manual hippocampal volume converters	19	79% (15)	21% (4)
Visual assessment non-converters	82	32% (26)	68% (56)
Fischl and Dale non-converters	82	30% (24)	70% (58)
Manual hippocampal volume non-converters	82	46% (38)	54% (44)

Results for fixed specificity	Number	MCI-c as AD	MCI-s as CTL
Visual assessment	101	68%	68%
Fischl and Dale	101	79%	68%
Manual hippocampal volume	101	68%	68%

AD = Alzheimer's disease, MCI = Mild Cognitive Impairment, CTL = healthy control, MCI-c = MCI converters and MCI-s = MCI stable. To better compare the performance of the three methods we also calculated the sensitivity (MCI-c predicted as AD) at a fixed specificity (MCI-s predicted as CTL). We set the specificity to that of the visual rating assessment and recalculated the sensitivity and specificity of the other two methods used.

## Discussion

Automated computerized MRI methods to aid in the diagnosis of AD will only be implemented in clinical practice if they are carefully investigated and validated. The aim of this study was to further validate the OPLS technique with fully automated MRI measures as input and compare it to the Scheltens scale for visual assessment of medial temporal lobe atrophy and to that of total hippocampal volume. To our knowledge this is the first time that the Scheltens visual rating scale has been compared to a computerized method. We wanted to investigate which approach would distinguish between AD patients and controls with the highest accuracy and best predict conversion from MCI to AD. We have previously shown that combining a set of automated measures of the brain together with manual measures of hippocampus significantly improves the prediction accuracy using OPLS [6]. Manual measures of different brain regions are time consuming and operator dependent however and are hence not regularly used in a clinical settings. Therefore we further investigated the power of OPLS using only automated measures as input (using the same volumes and cortical thickness measures as used here) in two large cohorts (AddNeuroMed and Alzheimer's disease Neuroimaging Initiative (ADNI)). This was performed to investigate if similar patterns of atrophy and prediction accuracy could be obtained from two different large cohorts using the OPLS model [9] and we found good comparability between the two cohorts. We have also previously investigated the value of combining magnetic resonance spectroscopy (MRS) with automated regional MRI measures using OPLS [7] which showed a significant improvement compared with using either set of measures individually. The next natural step was to compare the OPLS technique with a well established visual rating assessment scale such as the Scheltens scale, performed by an experienced neuroradiologist, as we describe here.

### Classification accuracy of the different techniques

The results suggest that the OPLS technique with fully automated regional MRI measures as input performs better than the visual rating assessments made by an experienced neuroradiologist. The sensitivity of the two methods is similar resulting in identical negative likelihood ratios (77.3%, 0.26 and 78.7%, 0.26 respectively), while the specificity was higher for the OPLS technique than the visual assessment (87.7% and 82.7%) yielding a higher positive likelihood ratio (6.3 and 4.6). The overall accuracy was higher for the OPLS technique compared to the visual rating scale (82.7% and 80.8%). When the specificity was fixed for all three methods at the value of the visual rating assessments, the sensitivity of the OPLS analysis (79%) was better than the other two methods (both 68%). Although the manually measured total hippocampal volume still yielded the best prediction accuracy, the time consuming nature of manual measures makes them impractical in a clinical settings. Manual measurements can also be operator dependent and it can be hard to compare such measures across sites and within sites if different operators are used. If only positive likelihood ratios are considered then the OPLS method with fully automated image measures performs as well as the manually measured total hippocampal volume. The results from the OPLS method using the fully automated MRI measures and the results from the hippocampal volumes are in line with our earlier results from the AddNeuroMed cohort and have been discussed and compared to that of other groups previously [6], [9]. Comparing the results from the current study with previously published work we found only one similar study which compared a computerized technique (SVM) with neuroradiologist evaluations [8]. In this prior study, two small cohorts of AD patients and controls were evaluated (40 subjects in the first cohort and 28 subjects in the second cohort). The SVM and visual assessments gave prediction accuracies of 95% vs. 88.8% respectively, for the first cohort, and 92.9% vs. 82.5% for the second cohort. The SVM results were better than the OPLS results that we report here for both cohorts, with the result from the visual rating assessment results higher for their first cohort than our study, but similar to our study for their second cohort. The cohorts included in the study by Klöppel et al. were neuropathologically confirmed and much smaller than the cohort investigated in the present study, which is a likely explanation for the higher prediction accuracy. Klöppel et al. also published another paper [5] using pathologically confirmed data to distinguish between AD and CTL using SVM. High prediction accuracies were again obtained, up to 96% but the sample sizes were again smaller (maximum 20 in each group). They also had a slightly larger cohort (33 AD and 57 CTL) with probable mild AD subjects. Using no feature selection, they obtained a prediction accuracy of 81.1% (sensitivity 60.6% and specificity 93.0%). Prediction accuracies can sometimes be misleading, especially if one group is much smaller than the other. In this present study using the OPLS method with no feature selection on the automated measure, we found an accuracy of 82.7% (sensitivity 77.3% and specificity 87.7%). As can be observed the accuracy of the latter study by Kloppel et al is very similar to ours, but their sensitivity is much lower. It is important that computerized analysis techniques are validated in large cohorts, since it is easy for such methods to fix onto features that may be different between small cohorts, but are not generalizable to larger cohorts. Although neuropathologically confirmed data is preferred when evaluating these types of automated models, it is very difficult to obtain large neuropathologically confirmed datasets in practice. This is even more pronounced when studying the prodromal stages of the disease. MCI subjects are typically diagnosed approximately 10–15 years before death, which makes longitudinal follow-up very difficult and the acquisition of large neuropathologically confirmed datasets with recent MRI an exceedingly difficult endeavor. At the moment it is necessary to choose between small data sets which are neuropathologically confirmed, but potentially not representative of the heterogeneity and complexity of Alzheimer's disease, and larger datasets such as ours, which are more representative but not neuropathologically confirmed. A further advantage of our study, compared to that of Klöppel et al. is that the images acquired in our study are ADNI compatible, making our findings generalizable to other large ADNI compatible cohorts.

The new research and diagnostic criterion have a strong focus on the use of biomarkers including MRI, PET and CSF [2], [3]. Although individual markers such as MRI can be powerful in AD, we believe that combining different markers is an attractive approach, particularly if considering differential diagnosis amongst different forms of dementia. Neurodegeneration in AD is estimated to start 20–30 years before the clinical diagnosis is given [47] and so if diagnosis is to be made at the prodromal stage of the disease then it may be necessary to combine several different markers of disease. Previously we have shown that combining regional MRI measures with magnetic resonance spectroscopy results improves classification results, doubling the positive likelihood ratio [7].

### Predicting conversion from MCI at baseline to AD at one year follow-up

At one year follow-up 19 of the 101 subjects with mild cognitive impairment converted from MCI to AD while 82 remained stable. One of the aims of this study was to compare how the three methods performed in predicting conversion from MCI to AD. The accuracy of predicting conversion varied from 68–79%. Total manual hippocampal volume gave the best results, followed by the OPLS approach including automated MRI measures and finally the visual rating assessment. It is however difficult to confidently state that one method is better than the other due to the small numbers of subject converting. The three methods predict 15/19, 14/19 and 13/19 converters respectively as more AD like, which is a small absolute difference that has a larger impact on the percentage accuracy. The accuracy of the different models in correctly predicting stable MCI subjects varied between from 54–70%. Although the OPLS method gave the best results, subjects diagnosed at baseline with MCI who are still classified as MCI at one year follow up may subsequently convert to AD at a later stage and thus assessing whether subjects will eventually convert to AD may require longer follow up times. Further large studies with longer follow up times are warranted to investigate this issue. To make the comparison of the performance of the three methods easier we calculated the sensitivity (MCI-c predicted as AD) at a fixed specificity (MCI-s predicted as CTL). This yielded the best sensitivity for the OPLS model with automated regional MRI measures as input. This model predicted 79% of the MCI-c subjects who converted to AD at one year follow-up as more AD-like, compared to 68% of the other two methods. Although the number of converters is relatively small, a likely explanation for these results is that combining multiple regions across the brain aids in the prediction of MCI conversion.

### Conclusion

Visual rating assessment of the medial temporal lobe gave similar prediction accuracy to computerized multivariate classification and both accuracies are comparable to that of manual hippocampal volume measurements. While manual hippocampal volumes are a valuable research tool and have found utility in clinical trials, they are not however practical for use in routine clinical work. Our results demonstrate that computerized multivariate classification is as good as expert radiological review of MRI using a validated and widely used visual rating scale, which suggests a potential future role for computerized methods as a complement to clinical assessment. Improving classification models by adding other biomarkers will hopefully increase the predictive power of the models and improve our understanding of the etiology of the disease. Two limitations of the current study are however that the data is not neuropathologically confirmed and that the follow-up of the MCI subjects was for one year.

In conclusion we believe that this study and previous work [6], [7], [9] has shown that the OPLS model with automated MRI measures as input has the potential to serve as a complement to clinical assessment of AD, and to target appropriate populations for clinical trials.
